# The Role of Intraperitoneal Carboplatin as Consalidation Chemotherapy in Women with Ovarian Carcinoma: Report of Our Experience and Systematic Review

**Published:** 2016-12

**Authors:** Azamsadat Mousavi, Mojgan Karimi-Zarchi, Nadere Behtash, Mitra Modares-Gilani, Mahnaz Mokhtari-Gorgani, Nili Mehrdad, Mitra Rouhi, Pouria Yazdian Anari

**Affiliations:** 1 Associate Prof, Gynecologist Oncologist, Tehran University of Medical Science, Iran; 2 Associate Prof, Gynecological Oncology Fellowship, Shahid Sadoughi University of Medical Science, Yazd, Iran; 3 Prof, Gynecologist Oncologist, Tehran University of Medical Science, Iran; 4 M.D, Obstetrics & Gynecologist, Tehran University of Medical Science, Iran; 5 Gynecologist Oncologist, Gynecological Oncology Fellowship, Tehran University of Medical Science, Iran; 6 Medical Student, Azad University of Medical Science, Yazd Branch, Iran; 7 Medical Student, Faculty of Medicine, Shahid Sadoughi University of Medical Sciences, Yazd, Iran

**Keywords:** epithelial ovarian cancer, consolidation chemotherapy, carboplatin, intraperitoneal injection, survival rate

## Abstract

**Background:**

Epithelial ovarian cancer is the leading cause of death from gynecology malignancy. The aim of this study was to assess the role of intraperitoneal chemotherapy with carboplatin, as a consolidative treatment, in reducing relapse and increasing survival of patients in advanced epithelial ovarian cancers, as well as evaluation of its toxicity.

**Methods:**

This clinical trial was conducted on 30 patients with epithelial ovarian cancer in stages II-IV in Gynecology oncology department in Valiasr University Hospital, Tehran during 2005-2010. They were enrolled through non-random sequential selection. They divided into 18 cases as the intervention group (receiving intraperitoneal chemotherapy) and 12 patients as the control group (with only retrospective follow-up). The cases received 3 cycles of 400 mg/m^2^ intraperitoneal carboplatin every 21 days following intravenous chemotherapy. Mean survival of two and five years, progression-free interval, overall survival, relapse, demographic parameters, drug toxicities and pathologic types of cancers were coded in the two groups and compared using SPSS 14.

**Results:**

The mean ages of cases and controls were 52.4 ± 8.6 and 55.1 ± 11.5 years. The mean duration of relapse-free survival was 13 ± 8.6 months for the cases and 9.5 ± 4.3 months for the control patients (not statistically different, *P*>0.05). The mean overall survival for cases and controls were 39 ± 16.5 and 30.8 ± 16.2 months, respectively (no significant difference, *P*>0.05). The frequency of drug toxicities in the cases was 5.6%, and consisted of mild-to-moderate abdominal pain, nausea and vomiting.

**Conclusion:**

consolidation therapy with intraperitoneal carboplatin may not increase overall survival, reduce relapse rate or decrease mortality, though it does not induce considerable side effects.

## INTRODUCTION

Epithelial ovarian cancer is the leading cause of death from gynecology malignancy (1, 2). Different treatments have been applied in these patients such as operation, chemotherapy (intraperitoneal, neoadjuvant or maintenance) and radiotherapy (14-18). Also contraception with oral contraceptive pill is one of ways of prevention of epithelial ovarian cancer (19).

Intraperitoneal chemotherapy is used as the second treatment in patients with advanced epithelial ovarian carcinoma. This regimen is considered as a good option because of reasons below:

75% of ovarian cancers accompany with advanced intraperitoneal disease, 30 to 50% of patients responded to primary treatment including chemotherapy and operation, have relapses mainly intraperitoneally (14-18).

Most of the patients response to chemotherapy following debulking surgery, but unfortunately their short term response and clinical outcomes are unsuccessful (3).

One of maintenance therapies in epithelial ovarian cancer is intraperitoneal chemotherapy. Superiority of this treatment is due to reasons below:

Physiologic and anatomic characteristics of peritoneum, high local concentration, longer contact with tumor and less toxicity in comparison with systemic therapy.

The patients with smaller tumor (microscopic size) or small macroscopic residue following the primary chemotherapy plus operation take more benefits.

Medications used for intraperitoneal therapy are cisplatin, carboplatin, etoposide, mitoxantrone, paclitaxel, topotecan and gemcitabine (3).

The aim of this study was to assess the effect of maintenance therapy with intraperitoneal carboplatin in advanced ovarian epithelial carcinoma. The main reason to do this study was due to high relapse rate of this tumor and lack of standard maintenance treatment.

## MATERIALS AND METHOD

This retrospective study in control group (checking documents of patients during years 2005 to 2010) and clinical trial one in case group (patients with stage II to IV epithelial ovarian cancer in gynecology oncology department) in valiasr university hospital of Tehran.

In this clinical trial, 30 patients who underwent complete operation without intestinal laceration complication and received 6 standard cycles of intravenous paclitaxel and carboplatin enrolled in this study. Imaging and tumor markers of them were normal. They were enrolled through non-random sequential selection.

18 cases as the intervention group (receiving intraperitoneal chemotherapy) and 12 patients as the control group (with only retrospective follow-up) were enrolled in our study.

The cases received 3 cycles of 400 mg/m^2^ intraperitoneal carboplatin every 21 days following intravenous chemotherapy. Follow up was done through documents in oncology department and phone contact with the patients or their families. demographic parameters, Mean survival of two and five years, progression-free interval, overall survival, drug toxicities, pathologic types of cancers and Relapse of disease (increasing or even doubling CA125 serum titer during one month, or any CA125 above 100 IU) or an abdominal or pelvic mass in ultrasound or physical exam in the two groups were coded and compared using SPSS 14. Any *P*<0.05 was considered as a significant difference.

### Findings

Mean age of patients was 52.4 ± 8.6 and 55.1 ± 11.5 years in case and control groups respectively. Also, mean duration without relapse was 13 ± 8.6 and 9.5 ± 4.3 in cases and controls respectively.

**Figure 1:**
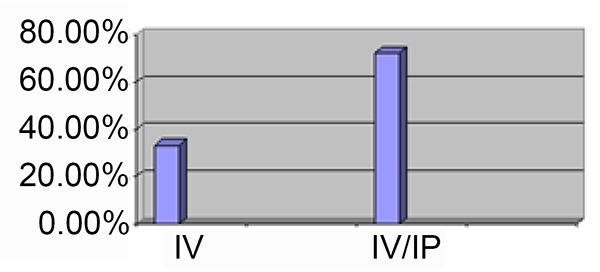
Survival rate (from the diagnosis until death): maintenance (Intravenous and Intraperitoneal chemotherapy) and follow up (only intravenous chemotherapy).

**Figure 2:**
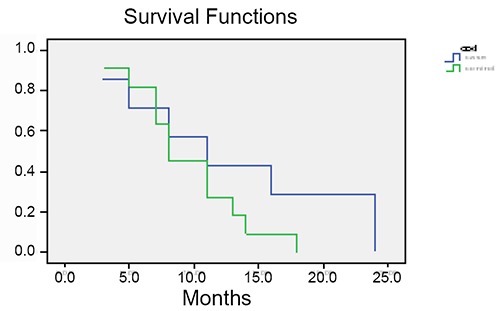
Progression-Free interval (from completing treatment until relapse) in both groups.

**Figure 3:**
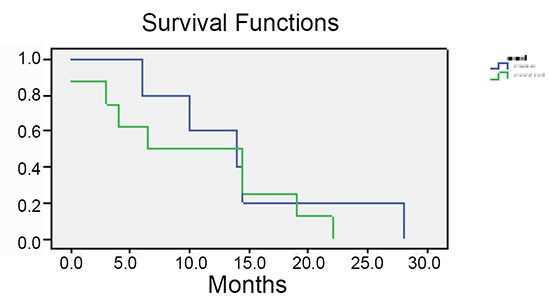
Survival rate in both groups.

Mean of survival rate was 39 ± 16.5 and 30.8 ± 16.2 months in case and control groups respectively. Five-year survival rate was 72.2% in patients received maintenance intraperitoneal chemotherapy with carboplatin, while it was 33.3% in controls (Figure 1, Figure 2, Figure 3).

It should be noted that no significant difference was seen in parameters above (*p*>0.05). However; toxicity rate was 5.6% in patients on maintenance therapy. Patients without relapse and those who stayed alive were not significantly more in case group than controls (*p*>0.05).

## DISCUSSION

In current study, total survival rate was 72.2% and 33.3% in case and control groups respectively. Mean of survival rate was higher in patients received 3 cycles of at least 400 mg/m^2^ intraperitoneal carboplatin than cases, but the difference was not significant. (39 ± 16.5 vs. 30.8 ± 16.2 months) (*P*<0.05).

In patients with epithelial ovarian carcinoma, first choice of treatment is operation. Purpose of operation is to confirm diagnosis, distribution of the disease and resection of visible mass. Intraperitoneal chemotherapy is not associated with systemic complications (4). It is recommended as the first line of therapy in advanced epithelial ovarian carcinoma (5).

Fujiware study showed that cisplatin can’t be used as a standard treatment due to its toxicity, so carboplatin was recommended as an alternative intraperitoneal chemotherapy (6).

Milczek reported the results of ovarian cancer treatment, where a regimen of intravenous cyclophosphamide followed by intraperitoneal cisplatin or carboplatin was administered as second line treatment. The patients divided into two groups, receiving 4 or 6 courses of treatment. There were 34% with complete and 31% with partial response, while 35% developed progressive disease. Median survival from the initiation of intraperitoneal chemotherapy (IP) was 51 months and significantly longer for patients who received four cycles of IP. They found that IP can be used in second line treatment of ovarian cancer, but six treatment cycles appear associated with worse results compared to four (7).

Bae *et al* evaluated the efficacy and feasibility of treating advanced ovarian cancer with paclitaxel or carboplatin in intraperitoneal hyperthermic chemotherapy (IPHC) during secondary surgery. Their results showed in stage III diseases, 5-year survival rates were 84.6% in IPHC-paclitaxel, 63.0% in IPHC-carboplatin and 32.8% in control group.

Three-year progression-free survival rates in stage III diseases were both 56.3% in IPHC-paclitaxel and IPHC-carboplatin and 16.7% in control group.

In advanced ovarian cancer, IPHC using paclitaxel or carboplatin during secondary surgery could be a candidate for regional consolidation therapy to prolong survival and hinder disease progression.

In this study, although patients without relapse were seen more in case group than control group, but the difference was not significant due to limited number of samples, so more patients and longer study is recommended to achieve better results (8) (Table 1).

**Table 1:** Clinical parameters: student t-test

Criteria		Total	Maintenance therapy	controls	*P*

Pathology	Serous cyst adenocarcinoma	7 (23.3%)	6 (33.3%)	1 (8.3%)	0.203
	Papillary serous adenocarcinoma	21 (70%)	11 (61.1%)	10 (83.3%)	
	Mucinous cyst adenocarcinoma	1 (3.3%)	1 (5.6%)	0 (0%)	
	Endometroid adenocarcinoma	1 (3.3%)	0 (0%)	1 (8.3%)	
Stage	1	2 (6.7%)	2 (11.1%)	0 (0%)	0.110
	2	3 (10%)	3 (16.7%)	0 (0%)	
	3	20 (66.7%)	9 (50%)	11 (91.7%)	
	4	5 (16.7%)	4 (22.2%)	1 (8.3%)	
Grade	1	2 (6.7%)	2 (11.1%)	0 (0%)	0.451
	2	22 (73.3%)	13 (72.2%)	9 (75%)	
	3	6 (20%)	3 (16.7%)	3 (25%)	
Relapse	Yes	18 (60%)	7 (38.9%)	11 (91.7%)	0.402
	No	12 (40%)	11 (61.1%)	1 (8.3%)	
Outcome	Dead	13 (43.3%)	5 (27.8%)	8 (66.7%)	0.35
	Alive	17 (56.7%)	13 (72.2%)	4 (33.3%)	

Comparing some parameters such as age, duration without relapse and survival rate between cases and controls showed *P* value 0.466, 0.273 and 0.402 respectively. According to student t-test, *P*<0.05 was considered statistically significant.

The value of IP mitoxantrone was studied as consolidation treatment of ovarian cancer at second-look surgery by Dufour on 50 patients with Stages II-IV ovarian cancer. Consolidation treatment consists of 20 mg (total dose per cycle) IP mitoxantrone every 3 weeks for six cycles. The results showed that toxicity was limited to mild abdominal pain not requiring dose reduction.

With a median follow-up of 2 years, the 5-year predicted survival is 59.8%, and the disease-free survival (DFS) rate is 47.3%. Patients with no or microscopic residual disease after initial surgery had a better 5-year DFS rate (75.8%) than those with macroscopic residual disease (31.2%). IP mitoxantrone (20 mg/cycle) is feasible with an acceptable abdominal toxicity (9).

Barakat study on 3 courses 100 mg/m^2^ intraperitoneal cisplatin + 200 mg/m^2^ etoposide as maintenance treatment in patients completing 1^st^ line treatment with no pathologic symptoms in 2^nd^ look, free-disease survival rate was significantly higher. The only significant predictors of long-term survival were grade and size of residual disease at initiation of IP therapy.

The median survival from initiation of IP therapy by residual disease was none, 8.7 years; microscopic, 4.8 years; less than 1 cm, 3.3 years; more than 1 cm, 1.2 years (10).

Another study done by Tournigand on patients with advanced ovarian cancer received every 4 weeks three consolidation cycles of IP chemotherapy (mitoxantrone, cisplatin, etoposide) showed that the median progression-free survival for the whole population was 34 months, 34% of the patients were estimated to be free of disease at 5 years. The median overall survival was 73 months, and the 5-year survival was 58% (11).

In EORT-55875 study done by Piccart, maintenance treatment consisted of 100 mg/m^2^ intraperitoneal cisplatin every 3 weeks for 4 courses. They found that mean of survival rate was 78 and 91 months in case and control groups respectively, which was not significantly different (12).

Fujiware recommended intraperitoneal carboplatin which was administered intravenously as one of the choices in ovarian cancer treatment.

According to pharmaceutics studies, intraperitoneal carboplatin not even reaches serum level as used intravenously, but also its 11-fold concentration in peritoneum in comparison with intravenous type is considerable. He evaluated the effect of intraperitoneal carboplatin as 1^st^ line treatment and estimated the proper dose of it as 400 mg/m^2^.

He also found that substitution of cisplatin with intraperitoneal carboplatin increase the tolerance of treatment. He reported that toxicity of carboplatin was so low and its complications were mainly associated with the catheter.

Although Fujiware is study was about 1st line treatment of ovarian cancer, but with respect to advantages of intraperitoneal carboplatin and lack of studies on maintenance therapy with carboplatin, it was recommended to do a study to evaluate the efficacy of maintenance therapy with carboplatin on advanced epithelial ovarian carcinoma.

In fujiware study, free-progress survival rate in patients on 5 courses of 400 mg/m^2^ or more and less than 400 mg/m^2^ intraperitoneal cisplatin was 51 and 52 months respectively (6).

According to previous studies, 5 courses of intraperitoneal cisplatin seem to be more useful which should studied more in future. On the other hand; free progress interval in patients on 3 cycles of at least 400 mg/m^2^ carboplatin was longer than those who didn’t receive IP chemotherapy. Although it was not significantly different, but clinically important and helpful to the patients.

According to similar studies, patients with advanced epithelial ovarian cancer who received intraperitoneal carboplatin had higher survival rates (5).

According to our findings, relapse rate was significantly lower in cases than controls. Also survival rate of patients on intraperitoneal carboplatin was higher significantly which was similar to previous studies.

In current study. Toxicity rate of taxol and carboplatin was reported 5.6% which was lower than previous studies. Similar studies reported 9.7% toxicity rate of IP/IV taxol and IP carboplatin, while in some other studies, 5-5.7% toxicity rate was reported (13).

It seems toxicity of carboplatin is less than others. On the other hand; toxicity is related to IV type of medication, so it is highly recommended to do more careful studies on IP carboplatin plus IV taxol toxicity.

In our study, Five-year survival rate was 72.2% in patients received maintenance intraperitoneal chemotherapy with carboplatin, while it was 33.3% in controls. The mean overall survival for cases and controls were 39 ± 16.5 and 30.8 ± 16.2 months, respectively (no significant difference, *P*>0.05), although 9 months can be clinically important and might be remarkable in studies with bigger samples size.

It seems that consolidation therapy with intraperitoneal carboplatin may not increase overall survival, reduce relapse rate or decrease mortality, though it does not induce considerable side effects.
